# When Evidence Fails to Change Practice: Examining the Persistence of Continuous Fetal Monitoring

**DOI:** 10.1177/10497323251347137

**Published:** 2025-06-09

**Authors:** Raymond G. De Vries, Lisa Kane Low, Meagan Chuey, Samia Abdelnabi, Maryn Lewallen

**Affiliations:** 1 Center for Bioethics and Social Sciences in Medicine, 1259 University of Michigan, Ann Arbor, MI, USA; 2 Department of Learning Health Sciences, 1259 University of Michigan, Ann Arbor, MI, USA; 3 Department of Obstetrics & Gynecology, School of Medicine, 1259 University of Michigan, Ann Arbor, MI, USA; 4 CAPHRI School for Public Health and Primary Care, 5211 Maastricht University, Maastricht, The Netherlands; 5 Department of Health Behavior and Biological Sciences, School of Nursing, 1259 University of Michigan, Ann Arbor, MI, USA

**Keywords:** fetal monitoring, intermittent auscultation, evidence-based practice, (de)implementation, perinatal quality improvement

## Abstract

Using scientific evidence to guide medical practice seems self-evident but, in certain specialties, it has proven difficult to realize. Use of continuous electronic fetal monitoring (cEFM) during labor is a case in point: research has shown that when compared to intermittent auscultation (IA), use of cEFM in uncomplicated labors of healthy women offers no clinical benefit and may result in unneeded interventions, and yet it remains common practice in obstetric care. In this study, we used observations on a labor and delivery unit and interviews with key informants to investigate the factors that drive the use of cEFM in the face of contrary evidence. Our observations of clinician behaviors regarding the use of cEFM and documentation of the effect of unit workflow on decisions about monitoring allowed us to identify several factors that drive the non-evidence-based use of cEFM. These include fear of liability, training, hospital unit policies, perceptions of patient desires, and workflow on the unit. What we learned about the continued use of cEFM offers insight into other instances where evidence fails to be implemented in practice. Our recommendations for how to align fetal assessment during labor with research evidence include more and better education about modes of fetal assessment for expectant parents and clinicians, hospital policies that encourage reflection on research evidence when making clinical decisions, attention to the way policies and protocols discourage use of IA, and making visible the clinical and economic benefits of evidence-based practice.

## Introduction

*You do continuous monitoring because that’s what we do, that’s the tradition.*

Director of Nursing on an obstetrics unit

By now the enigma of fetal assessment is well known. A large body of evidence—summarized in a Cochrane review (Alfirevic et al., 2017) and a recent meta-analysis (Al Wattar et al., 2021)—shows that when compared with intermittent auscultation (IA, listening to the fetal heartbeat at prescribed intervals), the use of continuous electronic fetal monitoring (cEFM) in uncomplicated labors with healthy pregnancies does not reduce rates of perinatal death or cerebral palsy and is associated with significantly increased risks of surgical and instrumental births. And yet, in United States, upwards of 85% of those in labor are monitored with cEFM (Declercq et al., 2013; Sakala et al., 2018).

Cesarean surgery can be a life-saving procedure, but it is not without risk. Compared to a vaginal birth, cesareans are associated with higher rates of postpartum maternal death, infection, venous thromboembolism, abnormal placentation, and uterine rupture (Molina et al., 2015). Following a first-time cesarean birth, maternal and neonatal risks increase substantially with each subsequent cesarean surgery (Silver, 2012). Additionally, cesarean births are more expensive than vaginal births and in-hospital recovery time is typically longer (Moran et al., 2020). Efforts to reduce surgical birth in healthy—sometimes referred to as “low-risk”^1^—pregnancies consistently describe how variation in the interpretation and use of fetal heart rate (FHR) tracings provided by cEFM drive the use of non-evidence-based (i.e., not medically indicated) cesarean surgery.

Acknowledging that cEFM may offer limited or no clinical benefit when used during healthy labors, the three major associations of maternity care professionals in the United States—the American College of Obstetricians and Gynecologists (ACOG), the American College of Nurse-Midwives (ACNM), and the Association of Women’s Health, Obstetric and Neonatal Nurses (AWHONN)—have cautioned their members about the routine use of cEFM for low-risk pregnancies. For example, an ACOG practice bulletin states, “Despite the frequency of its use, limitations of EFM include poor interobserver and intra-observer reliability, uncertain efficacy, and a high false-positive rate” (The American College of Obstetricians and Gynecologists, 2009, p. 2). The ACNM and AWHONN have published similar cautions, and first in the list of “Twenty-Five Things Nurses and Patients Should Question” published by the American Academy of Nursing is: “Don’t automatically initiate continuous electronic FHR (fetal heart rate) monitoring during labor for women without risk factors; consider intermittent auscultation (IA) first” (American Academy of Nursing, 2018, p. 1).

These evidence-based recommendations have proven difficult to realize in practice. Efforts to reduce the false-positive rate and the consequent harm of unnecessary cesareans have focused not on ways to reduce reliance on cEFM but on strategies to improve the reliability and accuracy of interpretation of FHR tracings. Most recently, computer analysis of FHR patterns has been tested as a strategy for reducing the potential for human error. A clinical trial of computerized decision support involving 47,062 women found that “use of computerised interpretation of cardiotocographs (i.e., fetal heart tracings) in women who have continuous electronic fetal monitoring in labour does not improve clinical outcomes for mothers or babies” (INFANT Collaborative Group, 2017, p. 1719). A systematic review and meta-analysis of the use of artificial intelligence (AI) in the interpretation of intrapartum FHR tracings found that using “AI and computer analysis for the interpretation of EFM during labor does not improve neonatal outcomes” (Balayla & Shrem, 2019, p. 7).

The question posed by the enigma of fetal assessment is: Why has a substantial body of evidence been unable to change practice? The statement we opened with offers a seemingly simple answer: “… that’s what we do, that’s the tradition.” The preference for tradition over evidence is an ongoing challenge, not only in maternity care (Hamm et al., 2023) but in all of health care (Hamm et al., 2023; Norton & Chambers, 2020). We need to understand why this is so: why do treatment traditions persist despite evidence to the contrary? If a practice is deemed ineffective, and especially if it *increases* risk, what can be done to eliminate it from routine care? A close look at why cEFM remains the standard of care—the “tradition”—in the face of contrary evidence (and guidelines based on that evidence) offers the opportunity to examine this question.

## Preparing to Enter the Field: The Historical Context and Stakeholder Perspectives on cEFM

In order to understand the gap between evidence and practice and recognizing the multiple dimensions of maternity care that come together to create the “traditions” of fetal assessment, we designed and initiated several studies that set the stage for this research. We started with the historical and ethical look at how courts respond in malpractice cases where medical evidence conflicts with common clinical practice (Spector-Bagdady et al., 2017). In the case of fetal assessment (as with other medical interventions), we found that when courts must choose between the best evidence and widely accepted practice, they most often side with how providers are commonly and currently practicing. When doctors accused of malpractice defend their actions by noting that they followed the evidence-based recommendations of their professional association, rather than the local “standard of care,” they often lose their case.

We next examined how expectant parents viewed the practice of fetal assessment. We looked at patient facing information about monitoring, the knowledge of new mothers about the risks and benefits of different approaches to monitoring, and women’s^2^ opinions about the need to provide consent for cEFM. Our content analysis of pregnancy-related websites and books found a paucity of accurate and accessible information that would allow expectant parents to make an informed choice about the type of monitoring to be used during labor (Torres et al., 2014). Information about IA was limited—more than one-third of the sources did not mention this approach to monitoring—and cEFM was often uncritically presented as the standard of care.

Our follow-up focus group study with 28 women who had given birth within the last two years confirmed the scarcity of information about cEFM. These new mothers knew very little about the risks and benefits of different modes of fetal assessment and expressed surprise that there were *any* risks associated with cEFM (Torres et al., Under Review). Noting that laboring women are rarely, if ever, asked to consent to the use of cEFM, we followed up with a survey asking women if they wanted the opportunity to choose, and give consent for, the type of monitoring used during labor. We found—perhaps not surprisingly—that more than 80% of respondents believed practitioners should obtain consent for monitoring (Dal Cin et al., 2021).

We then turned our attention to the providers of clinical care. We convened seven provider-specific focus groups with physicians (*n* = 7), nurses (*n* = 18), and midwives (*n* = 18) to solicit their experience with cEFM and their explanations of its persistence (Chuey et al., 2020). The participants were familiar with the evidence supporting IA for low-risk care and identified several factors related to the (over)use cEFM. These included demands of the clinical environment, hospital policies and procedures, patient influence, fear of liability, and the “deflection of responsibility” (i.e., the claim that it was another provider—not me—who insisted on the use of continuous monitoring). A more recent focus group study (Jepsen et al., 2022)—conducted with midwives—described a similar set of factors. They concluded that “the assigned roles of the cardiotocograph [i.e., the recording of the EFM] shape its everyday use more than evidence-based guidelines” (Jepsen et al., 2022, p. 1). The roles they identified include “babysitter, the midwives’ partner, an agent of shared responsibility, and a protector that ‘covers your back’” (Jepsen et al., 2022, p. 1).

Taken together, these findings begin to tell the story of the challenges of using evidence to alter the practice of fetal assessment. We learned that courts favor local “standards of practice” over evidence-based guidelines, that patients lack information, and that clinician behavior is shaped by the demands of the workplace. Nonetheless, these data are *reports* of what occurs on the maternity care unit, and not observations of *actual* behavior. It would be difficult, if not impossible, for example, to confirm the “deflection of responsibility” explanation—a key finding reported in our earlier paper (Chuey et al., 2020)—using only focus group data. Recognizing that “what we say” is not always “what we do” (Deutscher, 1973), we became observers on the unit, watching interactions between and among providers and patients related to modes of monitoring. To prepare for our observations and to clarify our unit observations, we organized a number of interviews with key informants. Our interviews and observations were designed to (1) observe clinician behaviors regarding the use of cEFM and IA, (2) document workflow processes involved in decisions about monitoring, and (3) gain insight into the effects of unit organization and culture on the use of IA and cEFM.

## Method

### Setting

We did our research in a busy perinatal care setting (commonly referred to as a labor and delivery unit) in a large academic medical center where approximately 4500 births occur annually. The hospital is a referral site for tertiary care and has a neonatal intensive care unit. The primary staff includes more than 300 nurses, 40 nurse-midwives, more than 70 OB/GYN faculty clinicians including maternal fetal medicine (MFM) providers, and an OB/GYN residency program with 24 residents. Family Medicine physicians also provide care and train residents on the unit. The nurse-midwifery service, established more than 40 years ago, provides continuity of care to patients who seek midwifery care. Nurse-midwives also serve as triage coordinators—seeing all patients who present in labor or with urgent care needs—and as laborists, working together with residents.

### Data Collection

Our interdisciplinary team included three nurse-midwives (LKL, MC, and SA), a public health researcher (ML), and a medical sociologist (RGDV), all of whom are experienced qualitative researchers. Three members of the team (MC, SA, and RGDV) spent a combined 121 hours on the unit between October 2018 and July 2019. Observations were done at different times of day for periods ranging from 1.5 to 5.5 hours, with a mean time of 3.56 hours. We shadowed nurses, midwives, family medicine physicians, and obstetricians and spent time at nurses’ stations, in triage, and in the common staff meeting room. Screens displaying the output of fetal monitors were present in all the locations we observed. Given the nature of the maternity unit, we kept written field notes during our observations—using the guidelines described by Phillippi and Lauderdale (2018)—transcribing them after leaving the field.

During our observations, we had numerous informal interviews with those working on the maternity unit. These were not recorded digitally but summarized in our field notes. Our observations and informal interviews were supplemented with formal, recorded interviews with six key informants who worked on the unit, done by two members of our team (LKL and RdV) between July and November 2019, guided by an interview script (see Appendix). Interviewees were purposefully selected because of their familiarity with, and opportunity to affect, policy and practice on the maternity care unit and within the Department of Obstetrics and Gynecology. Included were a Director of Nursing, an attending obstetrician, two maternal–fetal medicine specialists, a Director of Midwifery with administrative responsibilities within the unit, and a nurse (in training to be a midwife). During our time in the field, team members met regularly to examine our findings and adjust our observational strategies.

All interviewees provided written informed consent. All providers and birthing families on the unit were notified of our study via email and given the opportunity to opt out of being observed. Our study was approved by the University of Michigan IRB (approval number HUM00077024).

Our field notes and interviews were transcribed, checked for accuracy, and ML, in consultation with other team members, identified themes and subthemes in the data using MAXQDA software. Over the course of several team meetings, the (sub)themes were revised and were then used to write analytic memos that form the basis of the analysis presented here.

## Results

Like maternity care in other academic medical centers, the most frequently used method of fetal assessment during healthy labors was cEFM. Unit policy supported the evidence-based use of IA, but most patients received cEFM as the standard of care upon admission in labor, even when they were eligible for IA (Abdelnabi et al., 2021). When IA was used, it was primarily with women cared for by nurse-midwifery team members. Analysis of the data we collected via observations, conversations, and interviews revealed the choice of cEFM is driven by both clinical and non-clinical considerations. We begin with what we learned about the clinical drivers of continuous monitoring.

### The Clinical Value of cEFM

It is commonly assumed that the primary, if not the only, factor in the choice of how to assess the health of the fetus is the clinical situation of the laboring patient and the fetus. And indeed, clinicians routinely described the value of continuous monitoring for reducing the likelihood of harm. But they were also aware of the literature, reviewed above, that recommends against continuous monitoring for healthy or “low-risk” women. Their use of cEFM rather than IA was often framed as an effort to balance the perceived potential risk of harm to the fetus from intermittent monitoring against the risks of an unnecessary cesarean surgery resulting from a false positive of fetal distress noted via continuous monitoring. As one nurse put it:

We believe that … the risk … to the baby outweighs the risk of monitoring which … [means] that we’re more likely to have a C-section as an outcome. But the problem is you might be more likely to have a C-section as an outcome just because of the [focus on] risk to the baby.

But, as an obstetrician on the unit pointed out, not all risks are created equal. Referring to her experience with a mother whose baby died and had been monitored intermittently, she noted that when compared with the harm of an unnecessary cesarean section, a fetal death

… is so insurmountable. And looking at that mom and watching her watch her baby die, like you can’t ever help her emotionally recover from that. But I can help you emotionally recover from a mode of delivery that was not your preference. So, I think that although the studies show very rare instances where there’s benefit [of continuous monitoring] … the benefit that you get is so profound … And I think the thing that concerns me is that folks who are very pro intermittent monitoring sometimes brush off the rare badness.

A further complication in the risk balancing act is the lack of a clearly defined boundary between low and high risk. A nurse-midwife noted that the risk status of the mother and baby should guide the choice of fetal assessment method—that is, low-risk women should be monitored intermittently and high-risk women continuously—but, after acknowledging that fact, she asked:

… what does low-risk mean? [Our] criteria of what is considered high risk, or what is considered something we do interventions on, seems to encroach more and more into a range of what is normal. If we are very specific about what is low-risk, does that create such a narrow parameter where most of our patients don’t even meet that criteria?

Evidence-based recommendations that caution about the use of cEFM in healthy labors are easy to ignore when there is little or no guidance on how to define risk. It becomes even more difficult to follow those recommendations when they are tempered by the worry that intermittent monitoring may miss critical changes in the FHR offered by the minute-by-minute assessment of cEFM. This worry, expressed by several care providers, is clearly a clinical concern, but it also highlights a *non-clinical* reason for the use of cEFM. Yes, an unobserved change in the FHR may result in harm to a mother and baby but, in the legal environment of the United States, it also raises the specter of legal liability (Spector-Bagdady et al., 2017). Fear of being sued is a commonly cited reason for the overuse of cEFM: obstetricians will often say, “The only C-section you will get sued for is the one you *don’t* do.” Our research shows that this fear is just one among many non-clinical factors implicated in the inability of evidence to drive clinical practice.

### Non-Clinical Sources of Resistance to Evidence-Based Practice

The idea that non-clinical factors may be responsible for the decisions of caregivers runs counter to the common-sense notion that patient care is based on an evaluation of the clinical situation and the implementation of treatment based on the best evidence. Clinicians are, of course, keenly aware that non-clinical factors play a role in care delivery as evidenced by their frequent complaints about bureaucratic interference in their work (Alumran et al., 2024; Kruse et al., 2022; Thumm et al., 2022). In the case of fetal assessment, we found several non-clinical considerations that contribute to the use of continuous monitoring when intermittent monitoring is recommended.

#### Fear of Litigation

While fear of a malpractice suit may be the go-to explanation for overuse of cEFM, clinicians have a more nuanced view of how legal concerns affect the way fetal monitoring is done. In our conversations on the unit and in interviews, we heard “the elephant in the room that we don’t talk about a lot is … the fear of litigation,” but we also heard that liability was *not* the primary reason for the use of continuous monitoring. As one obstetrician said:

Sometimes people will demonize obstetricians and say like, “Oh, you’re just doing this to protect yourself so you don’t get sued.” But … I think that’s a little unfair characteristic because I think people … their main goal is that that baby’s not harmed, right?

Another obstetrician agreed but noted that doing a cesarean can, in fact, offer legal protection:

We don’t hopefully practice based on medical legal things and defensive medicine, but the reality is, is that I don’t know that obstetricians are getting sued for doing “unnecessary” C-sections. How are we going to define an unnecessary C-section? And anytime there’s a bad baby, the question always is, “Doctor, when could you have done a cesarean section?”

When asked if having an EFM tracing would protect a clinician from being sued, this same obstetrician, referring to several studies of the intra- and inter-rater reliability of the interpretation of EFM tracings (summarized in Hernandez Engelhart et al. (2023)), went on to say:

That actually doesn’t hold … because one of the challenges is that there is so much inter-observer variability and interpretation of the heart rate tracings … there’s a great study … where they actually took a group of “experts” and they gave them a clinical scenario, and they had the experts read the fetal heart rate tracings. Six months later, they gave the same group of experts the same tracings but changed the outcome. And if you change the outcome to an adverse outcome, then those doctors who previously said, “Oh, this was appropriate management,” said, “Oh no, they should have done [something else].” Exactly the same tracing. [The] same observer knowing the history, the hindsight bias, and looking through the “retrospectoscope.”

A colleague confirmed this view of the value of tracings in a courtroom:

… we all know that you can interpret strips in lots of different ways and almost anyone can find a way to interpret a good strip poorly, and vice versa. So, I think most people’s sort of knee jerk reaction is more monitoring is better and I’m not going to stop doing this because if I get sued I’m screwed.

When asked about the legal risk of cEFM versus IA, an obstetrician pointed out that those who work in academic hospitals have greater freedom to use IA because they are protected by the legal staff and the resources of their employer. Those in private practice must be more wary of the perceived risks of use of IA. This OB went on to say that when considering the value of IA, colleagues in California

brought some plaintiff’s attorneys to come in and talk to [them] about how they use different monitoring things and different strategies and how they use that to sue other doctors … if you’re going to engage lots of physicians in this thinking [about using IA], you have to bring the attorneys in and have that conversation about a plaintiff’s attorney, what do you look for? Does not having the strip, but having documented every 10 minutes normal heart tones by the nurse, is that more or less protective than having a strip?

We were also reminded that fear of litigation is not just about monetary loss:

… getting sued is not a trivial thing … there is a lot of emotional harm that comes from a lawsuit, it’s not just like I want to win. Your time is taken away, your emotional energy, your ability to care for other patients, your family, yourself is taken away. So, I think the system that we have in this country of lawsuits and blaming obstetricians for things that is not their fault is really problematic. And I think if we’re going to change how we provide obstetrical care, we need to include some reforms in that. … There is a lot of moral injury from unnecessary lawsuits.

It is difficult to disentangle the fear of harming your patients from the fear of being sued for that harm, but it is clear that cEFM has not alleviated anxiety in the labor room and that the fear of being sued reduces the use of IA, even when it is recommended as best practice.

#### Training and the Loss of Knowledge

Technologies, when widely adopted, can result in the loss of traditional knowledge and skills. This has been noted in the transition from vaginal to cesarean delivery of babies in a breech position (Glezerman, 2006, 2012): the publication of the now-challenged Hannah study (Hannah et al., 2000)—showing better outcomes for the use of cesarean surgery for breech presentations—resulted in the near disappearance of vaginal deliveries of breech babies. As a consequence, knowledge and skill required to manage a breech birth vaginally is gradually being lost.

The same is true in the case of fetal monitoring, where the possibility of cEFM has altered the education of the next generation of clinicians who no longer have the ability to assess the progress of labor and fetal status without the monitor. A nurse explained that reliance on cEFM has diminished the skills required to assess a woman in labor:

The strip is a piece of information, and the monitor is a piece of technology that’s no different than your hands. I think though what has happened over time is that people think that this thing [cEFM] is going to solve all their problems. This is going to tell them everything they need to know. And so I don’t have to rely on … my hands and, and what I know to be the right thing. And it’s hard to teach that stuff to people now because technology is everywhere.

In observing and talking with clinicians, we learned that training in IA is nearly non-existent. Commenting on her residency training, an attending OB told us:

We were not trained to do [IA]. We were very clearly told by our director of MFM that the benefits are very minimal. But he’s like, “But I want one of you to go manage a labor without a continuous fetal monitoring strip.” And he’s like, “You’re not going to do it.” And we were not taught how to do it. So, there was no intermittent monitoring at where I was training.

Medical students, doing clerkships on the unit we were observing, knew very little about IA. For example, a medical student who had been on the unit for five weeks was unaware of what intermittent monitoring was. Another medical student, doing a one-month visiting clerkship, was asked if he learned about IA in his training. He responded with a quizzical look and asked, “What is that?” When IA was explained, he recognized what it was and understood that cEFM, when used in healthy labors, could be a problem. He went on to make an interesting observation: “When you are a new resident,” he said, “you are uncertain of yourself and want all the information you can get. Continuous EFM provides that information, and early in your training you come to rely on cEFM, making it hard to stop using it.”

In our conversations, we heard about how the content and extent of training in fetal monitoring varied by provider type. Nurses are required to take an intermediate and an advanced course on fetal monitoring offered by AWHONN and must then complete a three-month orientation accompanied by an experienced nurse. On the other hand, residents learn these skills via a one-time four-hour course and by working with attending physicians. One resident, when asked how she learned to read “strips,” that is, the output of EFM, she mentioned “strip rounds” (an informal meeting of obstetrics staff where residents present and interpret EFM output), materials from ACOG, on-the-job training, and consulting with colleagues. Nurses expressed frustration that they are expected to maintain competencies in the interpretation of EFM strips, while physicians have no such requirement.

The apprenticeship model means that medical students and residents are limited to what they learn from their mentors and attending physicians, lessons that rarely include instruction in IA. In a discussion about IA and EFM, a nurse explained to her midwife colleague that she had talked with an attending physician who did not know that there was an agreed-upon definition for a “variable”—a shorthand reference for variable deceleration, an FHR change where the rate decreases, typically as the result of a temporary compression of the umbilical cord. The midwife noted that these were the doctors who are training residents and medical students, so if they don’t know the objective definitions associated with the interpretation of EFM tracings, then they obviously aren’t passing down that knowledge to others.

In describing their interaction with residents, two midwives commented on that lack of training. During her shift, one nurse-midwife got into a disagreement with a resident about the interpretation of a tracing: they could not agree on the baseline FHR—the general overall heart rate over time—and whether the patient was having accels or decels (acceleration/deceleration of the FHR). Like nurses, nurse-midwives found it frustrating that residents have little formal training in EFM. One nurse-midwife pointed out that residents take a four-hour class prior to working on the unit while nurse-midwives are required to be “EFM certified”—maintaining a biannual certification related to EFM interpretation. This variation in training creates the potential for variation in how fetal monitor tracings are interpreted.

In addition to disparities in training, some clinicians identified potential generational differences in support for use of cEFM compared to IA. More experienced nurses and nurse-midwives observed that younger colleagues—both nurses and MDs—tended to prefer cEFM. This attitude became apparent in a case described in our field notes where a patient, 32 weeks pregnant (40 weeks is the normal gestation period), came to the unit because she was having cramping. In an effort to assess the patient, a nurse-midwife was attempting to put the woman on a monitor for a 20-minute strip, but the machine was not working properly. Observing the scene, an experienced triage nurse commented that she prefers to do IA rather than EFM because it takes less time and is easier. She went on to observe that most nurses, especially the younger ones, rely more heavily on technology and are much more likely to prefer cEFM.

#### Policy

Unit policy has both direct and indirect effects on a provider’s decisions about the preferred mode of fetal assessment, promoting or discouraging evidence-based practice. Aside from official policies that endorse evidence-based use of IA, like those on the unit we observed, there are policies seemingly unrelated to monitoring, that nonetheless affect how fetal assessment is done. We found that the introduction of a new version of the electronic health record (EHR) resulted in a policy change that altered the way nurses were allowed to practice. The old EHR did not require a provider order for each phase of nursing care related to fetal assessment, giving nurses the freedom to make care decisions based on unit policy, practice guidelines, and the clinical situation. The new EHR restricted their autonomy in the care offered during labor:

With the [new system it] was very clear that we could not have protocols and policy outside of written orders … all of the stuff in the documents that [used to] guide nursing practice had to be written into the orders. [I] hear from nurses that they feel … like they have less autonomy to use their critical thinking. [It’s] also impacting any of the labor care that they’re providing. Over time, if you have to rely on going to the provider to get permission to do things and … you don’t know that person … you sort of stop going to them … you start saying, well they don’t want me to do this. [It’s] that progression thing. [It] was a big deal. I was really offended when we went [to the new system] because everything had to have an order.

On the other hand, the new EHR offered the opportunity to *encourage* evidenced-based practice. To save clinician time, the electronic record has pre-checked, default “order sets” for patients. A multidisciplinary joint practice committee—made up of midwives, obstetricians, and nurses—with the authority to create unit-based policies for all providers, saw this feature of the EHR as an opportunity to introduce evidence-based options for fetal assessment. Previously, the EHR for all patients admitted to the unit defaulted to an order for cEFM. The committee advocated to change the default admission order to intermittent fetal monitoring, requiring a clinician to provide a reason for using cEFM. A nurse on the committee explained:

… if you’re going to check the continuous monitoring, you have to select a risk factor in order to select that, and then everybody just by default is selected for intermittent monitoring. So, then people who are coming in for inductions or whatever they’re admitted for—whether it’s labor, induction—you’re not sitting on continuous fetal monitoring because maybe somebody hasn’t gotten to you [to see if cEFM is necessary].

Consistent with this nurse’s assessment, the change to a default that set IA as the standard led to an increase in the use of IA for appropriate candidates (Abdelnabi et al., 2021). This strategy has been effective in promoting the evidence-based use of IA at other institutions (Gams et al., 2019; Miller, 2020).

Hospital policies related to staffing also affect the choice of mode of fetal assessment. A nurse explains:

We talk about we’re a team, and you have to work in teams. … There is no way to birth a baby here on this floor without a team of people. The problem is, the team changes hourly, so it’s not a team inherently, it’s a group of people who keep coming together and you’re constantly forming and storming and that’s all you’re doing.

In an environment where the clinical team is constantly changing, the visual record offered by a tracing from cEFM becomes a much more practical way to share information than reports of what a clinician heard at the intervals of IA. As an obstetrician noted:

To use intermittent auscultation really requires a lot of trust between the doctor and the nurse, and the nurse has to be someone who that doctor has worked with previously, who is confident in their ability to assess things, trusts what they’re doing. Because a lot of times it’s just documented in their flow sheet that they’ve heard the fetal monitoring … A lot of people who take care of the low-risk women are younger nurses, people who just got hired. There’s almost 40 of us [MDs] who take calls for the low-risk labor patients. So, for those 40 people to build a trusting relationship with 200 nurses, that’s really hard. So I think to me that’s one of the challenges, is that I just have to believe that the nurse heard good heart tones and everything’s okay.

Routine, seemingly innocuous, policies can also generate overuse of cEFM. Like many OB units, the unit we observed required laboring women to have a non-stress test (NST) before admitting them. The NST is a test to measure the fetal heart rate and how it responds to movement and contractions using cEFM and typically lasting approximately 20 minutes. Once in place, however, it may become difficult to transition from cEFM to IA. A woman otherwise eligible for IA may be left on cEFM because, as noted above, it is not unusual to have varied interpretations of the tracings (Hernandez Engelhart et al., 2023) and it is simply easier to leave the monitor on than to advocate for transition to IA. It is a case of “guilty (of needing cEFM) until proven innocent (and thus eligible for IA),” abetted by the comfort that clinicians feel when a labor is continuously monitored.

#### The Role of the Patient

Our observations confirmed previous research that suggests patients often have limited knowledge of, and hence little influence on, the type of fetal monitoring used during their labors (Dal Cin et al., 2021; Torres et al., 2014, Under Review). To honor the privacy of patients and to remain consistent with HIPAA requirements and our IRB approval, we did not engage directly with patients. We did, however, ask providers about the role of patient preference in the type of monitoring used. One obstetrician said:

Well, I think right now it’s almost zero … I can excuse myself a little bit [because] most of the patients I see have some high-risk condition that opts them out of that. But that being said, not all of them do [have a high-risk condition] when they come into labor, and we are particularly bad at like eliciting patient preferences.

The obstetrician concluded:

But my hope is at this time [to have all caregivers ask] “How do you prefer to have your baby monitored?” And so I think right now very few of the physicians ask that question of patients and have a conversation. I think for most physicians, myself included, it is knee jerk to just do continuous monitoring and not to engage in a conversation and that's not how it should be done.

Another obstetrician noted the deference to professional expertise, saying that most patients will say, “Provide whatever you think is in the best interest of me and my baby.” This physician continued:

… the extremes get a lot of attention …. some of the high-risk patients … are like, “Don’t take my baby off the monitor. What if something happens?” I’m like, “Your baby is great, we can’t monitor you 24 hours a day, seven days a week” … the other extreme [is] where I’m really clinically worried about a patient scenario … what comes to mind is the example of the home birth transfer into the medical system that wasn’t desiring [cEFM] … she’s coming in with the predetermined mindset of I could have done this at home and I don’t want that monitoring. And that’s a big leap to make for her [when I] say I’m really worried about you and your baby’s wellbeing. But I think for the vast majority of patients, some of them will want to participate in that conversation, but many of them will just say, “You’re the expert.”

When, during interviews and observations, we asked about the role of patient preference, the most common answer was cEFM would be used, except in the unusual case where a patient asks for IA. Eliciting patient preference for type of monitoring was rare, as mentioned in our field notes:

Unless patient asks for IA or has it in birth plan, it seems like no one brings up patient preference or reviews it with her.

#### Workflow (Convenience)

Workflow refers to the everyday components that are considered part of creating an efficient operation of a maternity care unit: its formal and informal organization, the physical structure of the work floor, the allocation of responsibility for the care of laboring women, and the movement of women through the unit. In explaining the shift from IA to cEFM, an obstetrician pointed to “convenience and workflow”:

My interpretation of how things evolved is, frankly, a matter of convenience and workflow. It is much easier, I think, less work intensive, for the care provider to put someone on a fetal monitor, and you can go about your business and do another thing and the monitor’s basically doing its thing in the meantime … And the other part of convenience is that you don’t have to be at the bedside necessarily.

Several obstetricians noted how the evolution of the electronic monitor—from free-standing units in each patient room to central monitoring, where several patients could be watched remotely—reduced the workload of caregivers:

… when I first started, there was no central monitor[(ing)]. So even with a electronic fetal monitor, you had to be at the bedside to look at the strip coming out of the machine … So one of the, I think the sociological things [that happened] is this removed human beings from the bedside.

Our notes describe a conversation with an obstetrician about monitoring choice and workload:

[The OB] says, “cEFM is easier when you’re busy because at any point you can just look up and see a snapshot of what is going on at that time—you don’t have to track down the patient’s chart or scroll back on the monitor to view the heart rate.”

A nurse-midwife agreed that a change to IA will create more work:

… with IA, you definitely, I think, are at the bedside more which I think is a good thing for a lot of reasons, but I think will meet resistance from that aspect too, because it will change someone’s workflow who maybe has been doing a certain thing for a long time and now this feels like more work.

Clinicians who lived through the switch from in-room to central monitoring expressed concern that the convenience offered by the ability to “watch” your patients from a remote location came at the cost of losing bedside support for patients. Reflecting on research that shows the value of the face-to-face support offered by doulas for reducing unneeded intervention in labor and increasing Apgar scores (Fortier & Godwin, 2015), an obstetrician pointed out that while cEFM benefits clinicians, the more time-consuming use of IA may be more beneficial for patients:

And with continuous monitoring … everyone can sit in the board room [the central location displaying all the monitors] and be like, “Yep, everything’s fine.” Keep typing on my computer. And then doctors can do that and the nurses can do that and nobody actually has to go in and interact with the patient. And I think that part of me wonders if [with IA] … you just get more care in labor and more like emotional support, that everything’s okay and I’m checking in on you, and I’m making sure you’re okay from intermittent auscultation that you don’t get from continuous monitoring, because people can just ignore you.

Central monitoring affects the workflow of nurses in at least two ways: it makes it easy to “cross cover”—that is, share patient care—and it simplifies charting. Many times, on the unit we observed nurses who were sitting in spaces with central monitoring handing over patient coverage. Examples from our notes include:

One nurse to another: “Will you watch her while I’m gone?”—in reference to watching the nurse’s patient and the patient’s strip while she got something to eat.

Nurse: “Can you watch my baby? I need to go to my locker. She’s looking a little flat.”

Nurse Team Leader: “She’s probably just sleeping. She was a happy baby before.”

When a float nurse had to go to the bathroom she asked another RN, “Will you watch my shitty strip while I’m gone?”

Note how the word “watch” refers to keeping an eye on the central monitor output, rather than being in the room with the patient.

Continuous EFM also makes mandatory charting in a patient’s EHR over the course of labor easier for the nursing staff. If a nurse does not have time to update the patient’s EHR at scheduled time points or with the frequency required per the patient’s clinical status, it is possible to call up a patient’s cEFM tracing and scroll back to review and to report earlier outputs. The actual process of real-time charting then is hard to distinguish from retrospective charting done in this manner.

While a central monitoring station provides benefits to providers, not all clinicians value those benefits. A nurse-midwife told us she found the work required by IA to be a “gift”:

When I was a nurse, it was such a gift to have a patient who was on intermittent (auscultation), because you’re with your patient. You’re not messing with a monitor, you’re not readjusting the monitor, you’re not mashing on their belly …

## Discussion: The Interaction Between Themes

How is it that evidence supporting best clinical practice and the professional guidelines based on that evidence come to be ignored in everyday clinical work? The use of cEFM—a practice widely used despite compelling evidence that it has limited clinical value in the majority of labors and may cause unnecessary interventions—offers an ideal laboratory for answering this question. Our research found several factors that discourage the evidence-based use of cEFM, factors that likely play a role in other situations where evidence fails to change practice: fear of liability, training, unit policies, perceptions of patient desires, and workflow. Our dissection of the labor and delivery workplace presents these factors as discrete influences on the use of cEFM, but they are, of course, interconnected in ways that amplify their effect.

Fear of being sued for a poor outcome—a common explanation for over-dependence on cEFM—has several underappreciated downstream effects on clinical decision-making. An obstetrician worried about malpractice suits will limit the opportunity for the next generation of clinicians to be trained in the proper use of different modes of fetal monitoring. Absent knowledge of the risks and benefits of cEFM and IA, and lacking the skills required for IA, clinicians default to continuous monitoring. In settings where policy requires providers to specify the orders for fetal assessment, defaulting to cEFM means that IA will be underused, further diminishing nursing skills. Similarly, the workflow efficiency offered by cEFM contributes to its near universal use, making evidence-based guidelines for the use of IA difficult to implement and resulting in fewer people having the opportunity to do and learn IA, pushing cEFM as the default. This process serves to reinforce an approach to fetal monitoring not supported by evidence.

This pattern is also visible in how the physical space of a labor and delivery unit encourages non-evidence-based practice. The introduction of cEFM with its assumed, but untested, clinical value shaped the way OB units were designed and built, which, in turn, dictated the experience of laboring women (Wolf, 2018). The need for cEFM in every space where a laboring person may be present, together with the perceived value of central monitoring (Small et al., 2022a) that allows simultaneous observation of several patients by one clinician, meant that the laboring person was often confined to bed, tethered to monitoring apparatus, often leading to limited interaction with a clinician. Limited movement accompanied by limited support from unit staff can create discomfort that leads to requests for pain management. The typical response to this request is epidural anesthesia, a form of pain relief that requires cEFM, which in turn increases the demand for spaces with monitoring facilities.

Our findings align with recent research that shows factors that lead to the persistence of cEFM are complex and inter-related (Fox et al., 2024; Gottfreðsdóttir et al., 2025; Lamé et al., 2024; Small et al., 2021, 2022a, 2022b, 2023). Implicit in what we saw and heard on the unit is the fact that abandoning practices that have become routine is frightening. Clinicians fear harming their patients and the psychic, reputational, and legal harm that may follow. When research evidence is weighed against the evidence of clinical experience, the latter typically wins out, adding to the difficulty of changing practice. We suspect that other non-evidence-based medical practices are driven by these same factors.

## Conclusion

When a nurse told us, “You do continuous monitoring because that’s what we do, that’s the tradition,” she was revealing a deep truth about medical care. The dictionary definition of “tradition” (Merriam-Webster Dictionary, n.d) includes:

• an inherited, established, or customary pattern of thought and action;
• a belief or story or a body of beliefs or stories relating to the past that are commonly accepted as historical though not verifiable;
• the handing down of information, beliefs, and customs by word of mouth or by example from one generation to another without written instruction;
• cultural continuity in social attitudes, customs, and institutions; and
• characteristic manner, method, or style.

These five features of tradition are all present in the practice of fetal monitoring. The use of cEFM persists, not because the evidence shows it to be the best practice, but because it is the customary pattern of action, commonly accepted, handed down from mentors to students, aligns with the needs of institutions, and is the characteristic method of delivering maternity care. We surmise that what we learned about the continued use of cEFM can explain other instances where evidence fails to be implemented in medical practice.

What does our research mean for those who wish to see evidence replace tradition in the practice of medicine? The obvious but difficult lesson is that we need programs that wean clinicians off comfortable, but non-evidence-based practices. As we have seen, the organization and culture of the workplace, and not research evidence, often determine clinical practice.

With regard to cEFM, this research, together with our earlier work, suggests several changes—some easier than others—that would redirect practice in a more evidence-based direction:

• Better education about fetal monitoring for expectant families, including required consent, informed by research on the benefits and risks of cEFM and IA;
• Rethinking hospital policies—not just those specifically addressing monitoring, but those *related to how monitoring is deployed.* Consider, for example, how the requirements of an EHR, or mandatory NSTs on admission, or staffing policies can lead to cEFM throughout labor and discourage use of IA;
• Challenging the oft-made assumption that central monitoring is cost efficient (Sartwelle, 2012) and that implementing the national nursing staffing standard of one-to-one care (Association of Women’s Health, Obstetric and Neonatal Nurses, 2022) that supports the use of IA would increase costs; and
• Requiring formal and ongoing training on IA and cEFM for physicians, not just nurses and midwives.

We are not recommending *de-implementation* as much as we are recommending *implementing* practices that use what we know about monitoring to organize the everyday routines in maternity care. This is the first step in creating a tradition of evidence-based practice.

## Supplemental Material

Supplemental Material - When Evidence Fails to Change Practice: Examining the Persistence of Continuous Fetal Monitoring Supplemental Material for When Evidence Fails to Change Practice: Examining the Persistence of Continuous Fetal Monitoring by Raymond G. De Vries, Lisa K. Low, Meagan Chuey, Samia Abdelnabi, and Maryn Lewallen in Qualitative Health Research

## Notes

1. The use of the word “risk” is fraught. What some call “healthy” pregnancies, others call “low-risk,” implying ever-present danger, even when a pregnancy is proceeding normally, without complications. We use these two terms interchangeably, reflecting how different people involved in maternity care think about pregnancy and birth.

2. In recent years, use of the word “woman” for those who are pregnant has become controversial because it excludes pregnant people who do not identify as women. On the other hand, to forgo use of the word woman makes invisible the identity of an overwhelming majority of pregnant people. In an effort to be inclusive without erasure, we use both woman/women and non-gender specific terms to refer to those who are pregnant.
