# Supplementary material for: When Evidence Fails to Change Practice: Examining the Persistence of Continuous Fetal Monitoring
Source: Qual Health Res. 2025 Jun 9;36(12):1298–310. doi: 10.1177/10497323251347137 (PMC13538909; doi:10.1177/10497323251347137)
Supplement: Supplementary file 1 [file sj-pdf-1-qhr-10.1177_10497323251347137.pdf]

Appendix

Interview script:

I will give you a few minutes to read the form.

Are there any questions about the consent form? [Respond to questions]

If there are no further questions, please sign and date the form. If you feel you cannot sign the form, then we will not conduct the interview. Thanks for considering being in our study.

<u>Script</u>

Again, thank you for taking the time to talk to me. I am <   > and this is <   >. I work at <   > and <   > works at <   >. As you saw in your invitation we are part of research team looking at the use of electronic fetal monitoring.

<u>Icebreaker</u>:

Let's begin by having you introduce yourself, if you can tell me about where you were trained, where you work, and what you most enjoy about your work.

Thanks. As you know, we are interested in learning more about the use of electronic fetal monitoring. Over the next few months we will be conducting several interviews with labor and delivery nurses, obstetricians, midwives, lawyers, and hospital administrators, supervisors and department heads.

We will be asking open-ended questions. There are no right or wrong answers to these questions. I am sure I do not have to say this, but please feel free to share your point of view, it will help us if you simply speak your mind.

There are some digital recorders here. We are recording the session because we don't want to miss any of your comments. People often say very helpful things in these discussions and we can't write fast enough to get them all down. We will not use names in our reports. When we transcribe these recordings, we will not include any names; when the transcriptions are complete the recordings will be destroyed. When we publish any of these data, names and places will not be used. Let's begin.

<u>Questions</u>

We are interested in learning more about the many reasons that clinicians use EFM as a routine part of the care given to women in labor. We are particularly interested in how your training, features of your work environment – including relationships with co-workers, institutional policies, aspects of technology, and attitudes of patients – and features of the legal environment influence your use of EFM. Let's consider each of these.

Using EFM

1. How do you use EFM in practice? Why do you choose to use EFM in this way?
    a. How has your training interacted with the common practices where you work now?

2. How does your work environment influence your use of EFM?
   Probes
    a. Explore 1) patterns of work, 2) division of labor and 3) the distribution of authority
    b. How does the technical environment influence your use of EFM?
        i. Central monitoring
        ii. Links to patient information systems

3. What role do patients play in the routine use of EFM?
   Probes
    a. What kind of conversations about EFM do you have with patients, and when do they occur (antenatally, or when patients are admitted to labor and delivery)?
    b. For what procedures do you seek specific consent from patients? How do you decide which procedures require consent?
    c. What happens when patients decline (or refuse) continuous EFM? How do you respond? How do other care providers (doctors/nurses, depending on what group it is) respond?
    d. What does your institution do to manage labor *without* continuous EFM?

4. How is your use of EFM influenced by the legal environment?
   Probes
    a. What do you know about the value of EFM in malpractice cases? That is, how do EFM strips protect or harm a clinician in court proceedings?

Interpreting EFM findings

5. Tell us about times when EFM helped you prevent a poor neonatal outcome (i.e. when EFM led to an urgent cesarean and the baby had poor cord gasses or poor Apgar's).
   Probes
    a. Tell us about times when EFM might have misled you, and caused you to do an urgent cesarean that perhaps wasn't ultimately needed in the time frame in which it was performed?
    b. What resources would help you in making decisions about how to use EFM to better manage labors?
    c. What would it take to come to shared and standardized approaches to interpreting EFM?

Familiarity with EFM research/sources of knowledge

6. There is a lot of information out there about the clinical value of EFM and about the consequences of EFM use. What do you know about this information?

    Probes

    a. What is the relationship between EFM and adverse birth outcomes?
    b. What is the relationship between EFM and cesarean section?

7. Where did you learn about the risks/benefits of EFM?

    Probes

    a. Residency, current institution, salespeople for electronic fetal monitors?
    b. How has your training interacted with the common practices where you work now?

8. How do you balance the findings of research with your use of EFM?

    Probes

    a. If there is dissonance between what the research says and how you use EFM, how do you harmonize/justify this difference?
    b. What strategies do you use to reduce dissonance?

And finally,

9. What did we miss? What did we *not* ask about the use of EFM that you think we should have asked?
